# Regulation of Expression of Cannabinoid CB_2_ and Serotonin 5HT_1A_ Receptor Complexes by Cannabinoids in Animal Models of Hypoxia and in Oxygen/Glucose-Deprived Neurons

**DOI:** 10.3390/ijms23179695

**Published:** 2022-08-26

**Authors:** Jaume Lillo, Iu Raïch, Laura Silva, David A. Zafra, Alejandro Lillo, Carlos Ferreiro-Vera, Verónica Sánchez de Medina, José Martínez-Orgado, Rafael Franco, Gemma Navarro

**Affiliations:** 1 CiberNed, Network Center for Neurodegenerative Diseases, National Spanish Health Institute Carlos III, 28040 Madrid, Spain; 2 Molecular Neurobiology Laboratory, Department of Biochemistry and Molecular Biomedicine, Faculty of Biology, Universitat de Barcelona, 08028 Barcelona, Spain; 3 Institute of Neuroscience, University of Barcelona (NeuroUB), Av Joan XXIII 27-31, 08028 Barcelona, Spain; 4 Molecular Neuropharmacology Laboratory, Department of Biochemistry and Physiology, School of Pharmacy and Food Science, Universitat de Barcelona, 08028 Barcelona, Spain; 5 Biomedical Research Foundation, Hospital Clínico San Carlos-IdISSC, 28040 Madrid, Spain; 6 Department of Neonatology, Hospital Clínico San Carlos-IdISSC, 28040 Madrid, Spain; 7 Phytoplant Research S.L.U, Astrónoma Cecilia Payne Street, Centauro Building, B-1, 14014 Córdoba, Spain; 8 School of Chemistry, Universitat de Barcelona, 08028 Barcelona, Spain

**Keywords:** downregulation, heteromers, hypoxia, ischemia, phytocannabinoids, serotonin

## Abstract

**Background:** Cannabidiol (CBD) is a phytocannabinoid with potential in one of the most prevalent syndromes occurring at birth, the hypoxia of the neonate. CBD targets a variety of proteins, cannabinoid CB_2_ and serotonin 5HT_1A_ receptors included. These two receptors may interact to form heteromers (CB_2_–5HT_1A_-Hets) that are also a target of CBD. **Aims:** We aimed to assess whether the expression and function of CB_2_–5HT_1A_-Hets is affected by CBD in animal models of hypoxia of the neonate and in glucose- and oxygen-deprived neurons. **Methods:** We developed a quantitation of signal transduction events in a heterologous system and in glucose/oxygen-deprived neurons. The expression of receptors was assessed by immuno-cyto and -histochemistry and, also, by using the only existing technique to visualize CB_2_–5HT_1A_-Hets fixed cultured cells and tissue sections (in situ proximity ligation PLA assay). **Results:** CBD and cannabigerol, which were used for comparative purposes, affected the structure of the heteromer, but in a qualitatively different way; CBD but not CBG increased the affinity of the CB_2_ and 5HT_1A_ receptor–receptor interaction. Both cannabinoids regulated the effects of CB_2_ and 5HT_1A_ receptor agonists. CBD was able to revert the upregulation of heteromers occurring when neurons were deprived of oxygen and glucose. CBD significantly reduced the increased expression of the CB_2_–5HT_1A_-Het in glucose/oxygen-deprived neurons. Importantly, in brain sections of a hypoxia/ischemia animal model, administration of CBD led to a significant reduction in the expression of CB_2_–5HT_1A_-Hets. **Conclusions:** Benefits of CBD in the hypoxia of the neonate are mediated by acting on CB_2_–5HT_1A_-Hets and by reducing the aberrant expression of the receptor–receptor complex in hypoxic-ischemic conditions. These results reinforce the potential of CBD for the therapy of the hypoxia of the neonate.

## 1. Introduction

Cannabidiol (CBD) is one of the most studied components of *Cannabis sativa* L. The compound is approved for human use and is attracting further interest due to possible additional health benefits in a variety of diseases/syndromes [1,2,3,4,5,6,7,8]. One important advantage over the most studied molecule in *Cannabis sativa* L., Δ^9^-tetrahydrocannabinol (Δ^9^-THC), is the lack of psychotropic effects. Like Δ^9^-THC, CBD was supposed to act through cannabinoid receptors. Despite it may act as an agonist by binding the orthosteric center of cannabinoid CB_1_ and CB_2_ receptors [9,10,11,12,13], the compound also modulates these two receptors in an allosteric fashion [14,15,16]. Its mode of action seems to be multifaceted as it has, among others, agonistic properties at serotonin 5HT_1A_ receptors and modulates GPR55, peroxisome proliferator-activated receptors and potential cation TRPV1 channels [17,18,19,20,21].

CBD at 1 mg/kg single dose reduces hypoxia/ischemia-induced brain damage in newborn rats, mice, and piglets by, among other factors, diminishing excitotoxic damage, inflammation, and oxidative stress [22,23,24,25]. More recently, neuroprotection by CBD in a neonatal rat model of perinatal arterial ischemic stroke (PAIS) allows for functional recovery by reducing neuronal death and astrogliosis, as well as by decreasing apoptosis and metabolic alterations and by reducing neuroinflammation [26]. In that work, CBD was administered shortly after the end of MCAO (middle cerebral artery occlusion), and the selected dose was 5 mg/kg [26]. However, PAIS symptoms in a newborn are usually subtle and unspecific, which often determines that PAIS is not diagnosed or is diagnosed several days after delivery, when stroke is likely occurring [27]. Therefore, it is necessary to find an effective treatment despite the fact that it begins hours after the PAIS. CBD has demonstrated a broad temporal therapeutic window in adult mice models of stroke [28]. There are no data on the therapeutic window of CBD in newborn rats after PAIS. However, CBD showed a therapeutic window between 18 and 24 h in a mouse model of hypoxic-ischemic brain damage in newborns [29].

G-protein-coupled receptors may lead to protein–protein interactions resulting in heteromers, whose properties are different from those displayed by monomeric receptors [30,31,32]. Some of the CBD targets can form heteromers; among others, GPR55 with cannabinoid CB_1_ or CB_2_ receptors and the 5HT_1A_ receptor with the cannabinoid CB_2_ receptor [25,33,34,35,36]. In addition, the pharmacological effects of CBD at cannabinoid receptors have been reported to depend on whether or not CB_1_-CB_2_ receptor heteromers are formed [37]. Interestingly, the benefits of CBD in preclinical models of hypoxic brain injury are mediated by CB_2_ and 5HT_1A_ receptors, the compound being able to prevent some of the behavioral consequences of carotid artery occlusion via, among others, CB_2_ and 5HT_1A_ receptors [38]. As the expression of heteromers formed by CB_2_ and serotonin 5HT_1A_ receptors (CB_2_-5HT_1A_-Hets) increases in the brain of a model of newborn hypoxic-ischemic brain damage [35], we here addressed how CBD affects receptor pharmacology and expression of these heteroreceptor complexes in glucose-oxygen-deprived (GOD) cells. Moreover, the expression of CB_2_-5HT_1A_-Hets was determined in GOD neurons. For comparison, another phytocannabinoid, cannabigerol (CBG), was incorporated into the study.

## 2. Results

### 2.1. In Vivo HI-Induced Brain Damage

Rats in different groups were similar in age (8.6 ± 0.3, 8.6 ± 0.2, and 8.7 ± 0.1 d for sham, HI + vehicle, and HI + CBD, respectively; *p* > 0.05) and weight (18 ± 2, 18 ± 2, and 18.9 ± 0.6 g for sham, HI + vehicle, and HI + CBD, respectively; *p* > 0.05).

HI led to brain damage, as assessed by MRI, which was reduced by CBD treatment (volume of damage: FR3 = 19.6 ± 0.5 vs. 16.2 ± 0.4% for HI + vehicle and HI + CBD, respectively, *p* < 0.05; FR2 = 22 ± 1 vs. 14 ± 1% for HI + vehicle and HI + CBD, respectively, *p* < 0.05; FR1 = 24 ± 5 vs. 10 ± 5% for HI + vehicle and HI + CBD, respectively, *p* < 0.05).

### 2.2. CBD and CBG Favour CB_2_–5HT_1A_ Receptor Complex Formation

**Figure 1:**
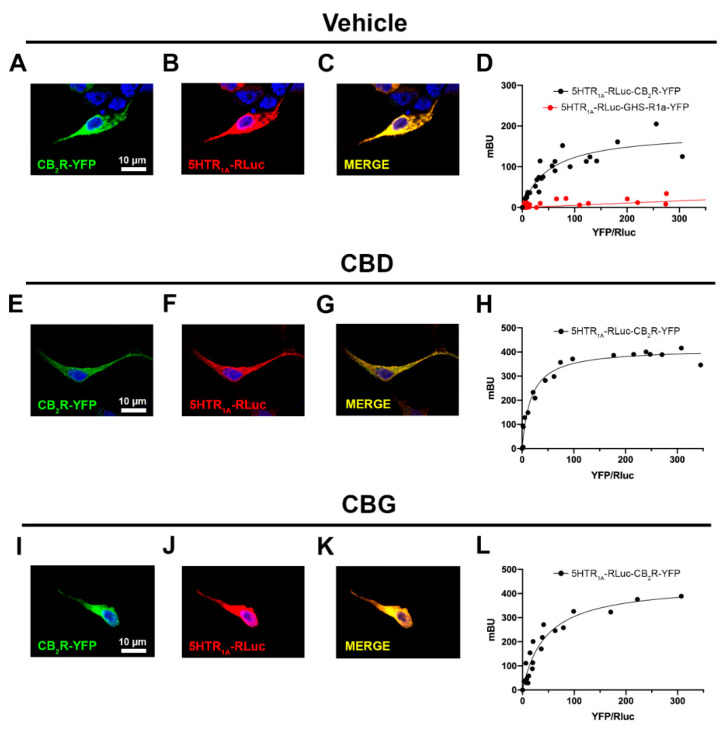
Effect of CBD or CBG on the 5HT_1A_R–CB_2_R interaction. (**A**–**C**,**E**–**G**,**I**–**K**) Confocal microscope images of HEK-293T cells expressing CB_2_R-YFP (0.75 μg cDNA) and 5HT_1A_R-RLuc (0.5 μg cDNA). Cells were pretreated with 200 μM of CBD (**A**–**C**), 200 μM of CBG (**E**–**G**), or vehicle (**I**–**K**) for 30 min. 5HT_1A_R-RLuc (red) was identified by immunocytochemistry using an anti-RLuc antibody. The CB_2_R-YFP (green) was identified by its own fluorescence. Co-localization is shown in yellow. Cell nuclei were stained with Hoechst (blue). Scale bar: 10 μm. (**D**,**H**,**L**) Bioluminescence resonance energy transfer (BRET) assays performed in HEK-293T cells co-transfected with a constant amount of cDNA for 5HT_1A_R-RLuc (1.5 μg) and increasing amounts of cDNA for CB_2_R-YFP (0.2 to 4 μg) or, as negative control, with a constant amount of cDNA for 5HT_1A_R-RLuc (0.75 μg) and increasing amounts of cDNA for GHSR-1a-YFP (0.2 to 2 μg cDNA). Transfected cells were pretreated with 200 nM of CBD (**D**), 200 nM of CBG (**H**), or vehicle (**L**) for 30 min before fluorescence emission was recorded. BRET data are expressed as the mean ± S.E.M of 8 different experiments performed in duplicates. mBU: milliBret units.

Due to the fact that 5HT_1A_ and CB_2_ receptors can interact to form CB_2_–5HT_1A_- receptor heteromers (CB_2_–5HT_1A_-Hets), we first set out to assess whether CBD affects the receptor-receptor interaction. For comparison, the effect of another relevant phytocannabinoid, cannabigerol (CBG), was also determined. Using immunocytochemical assays in HEK-293T cells that co-express the CB_2_R fused to YFP and the 5HT_1A_R fused to RLuc (Figure 1), it was observed that the receptors colocalize in plasma and intracellular membranes; the degree of colocalization is shown in yellow in Figure 1C.

In cells pre-treated (30 min) with CBD or CBG, no significant differences were observed on receptor expression and colocalization (Figure 1). We then performed BRET experiments using HEK-293T cells expressing a constant amount of 5HT_1A_-RLuc and increasing amounts of CB_2_R-YFP. Consistent with our previous results, a saturation BRET curve was obtained indicating interaction of the two receptors (BRET_max_ = 185 ± 19 mBU; BRET_50_ = 51 ± 14) to form CB_2_–5HT_1A_ receptor complexes (Figure 1D). Interestingly, the pre-treatment with 200 nM CBD notably increased the BRET_max_ (414 ± 13 mBU) and the apparent affinity (BRET_50_ = 17 ± 3), indicating that CBD increases the number of complexes formed and/or induces a structural reorganization of the CB_2_-5HT_1A_ receptor complex. Pre-treatment with 200 nM CBG increased the BRET_max_ (440 ± 40 mBU) without significantly affecting the BRET_50_ (44 ± 9) (Figure 1L). Cannabinoids did not affect receptor expression. As a negative control, HEK-293T cells expressing a constant amount of 5HT_1A_-RLuc and increasing amounts of GHS-R1a-YFP (Figure 1D) gave a linear signal indicating the lack of interaction between these two receptors.

### 2.3. CBD and CBG Blocked β-Arrestin 2 Recruitment Induced by Serotonin in Cells Expressing CB_2_–5HT_1A_-Hets

**Figure 2:**
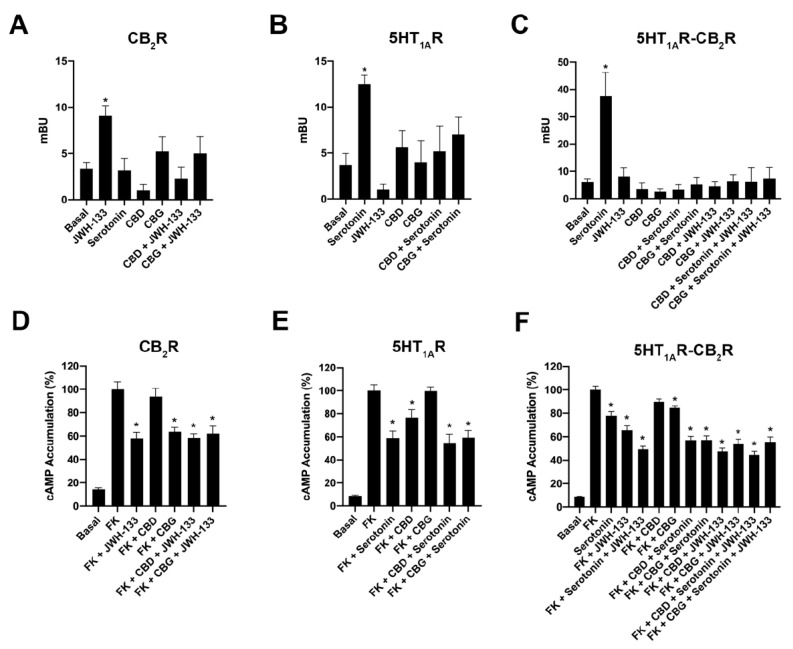
Effect CBD or CBG on the functionality of receptors expressed in HEK-293T cells. (**A**–**C**) β-Arrestin 2 recruitment assays were performed in HEK-293T transfected with cDNAs encoding for either CB_2_R-YFP (1.5 μg) (**A**), 5HT_1A_R-YFP (1.5 μg) (**B**), or CB_2_R (1.5 μg) and 5HT_1A_R-YFP (**C**). In all cases, β-arrestin 2-RLuc (1 μg cDNA) was also expressed. (**D**–**F**) Intracellular cAMP assays were performed in HEK-293T cells transfected with the cDNA encoding for either CB_2_R (1.5 μg) (**D**), 5HT_1A_R (1.5 μg) (**E**), or both (**F**). In β-arrestin 2 recruitment and cAMP experiments, cells were pre-treated with 200 nM CBD, 200 nM CBG or vehicle and subsequently stimulated with the selective agonists, 200 nM JWH-133 -CB_2_R- or 200 nM serotonin -5HT_1A_R-. After the treatment with the agonists, cAMP levels after 0.5μM forskolin stimulation were detected by the LanceUltra cAMP kit and the results were expressed as a percentage with respect to levels obtained upon forskolin stimulation. β-Arrestin 2 recruitment was determined 25 min after treatment in cells expressing CB_2_R (**A**) or 5HT_1A_R and CB_2_R (**C**), or 7 min after treatment in cells expressing 5HT_1A_R (**B**). The values are the mean ± SEM of 10 different experiments performed in triplicate. One-way ANOVA followed by Dunnett’s multiple comparison post hoc test were used for statistical analysis. * *p* < 0.05, versus basal condition in β-arrestin 2 recruitment experiments or versus 0.5 μM forskolin stimulation in cAMP assays.

After showing that CBD and CBG favor the formation of the CB_2_–5HT_1A_ receptor complex, we questioned their effect on receptor functionality. First, β-arrestin 2 recruitment was analyzed by BRET in HEK-293T cells expressing β-arrestin 2-RLuc and either CB_2_R-YFP, 5HT_1A_R-YFP, or CB_2_R-YFP and 5HT_1A_R. Results from experiments in CB_2_R-expressing cells showed that both CBD and CBG partially blocked the effect of the selective CB_2_R agonist, JWH-133 (Figure 2A). Similarly, both phytocannabinoids partially blocked the effect of serotonin in 5HT_1A_R-expressing cells (Figure 2B). When results obtained in cells expressing CB_2_–5HT_1A_-Hets were analyzed, it was first noticed that the effect of serotonin on recruiting β-arrestin 2-RLuc to the CB_2_R-YFP was marked, whereas the effect of selective CB_2_R agonist was negligible (Figure 2C). In those cells expressing the CB_2_–5HT_1A_-Hets, both CBD and CBG completely blocked the effect induced by serotonin.

Due to the fact that both CB_2_ and 5HT_1A_ receptors couple to G_i_ proteins, we performed cytosolic cAMP determination experiments after treatment with 0.5 µM forskolin in cells whose receptors were activated in the absence and presence of CBD or CBG. In cells expressing the CB_2_R, the selective agonist, JWH-133, produced a significant decrease in forskolin-induced cAMP levels (Figure 2D). Interestingly, CBG (200 nM) led to a similar decrease in forskolin-induced cAMP levels. The effect of CBD was not significant, and coactivation using JWH-133 and either CBG or CBD led to values such as those obtained using JWH-133 alone (Figure 2D). In cells expressing the serotonin 5HT_1A_ receptor, it was CBD, but not CBG, that induced a significant decrease in the cAMP levels induced by forskolin. Coactivation using serotonin and either CBG or CBD led to values similar to those obtained using serotonin (Figure 2E). Finally, in HEK-293T cells co-expressing CB_2_ and 5HT_1A_ receptors, both JWH-133 and serotonin produced a significant effect that was potentiated when the two compounds were added together. Interestingly, the action of serotonin, but not JWH-133, was enhanced by the two phytocannabinoids, CBD and CBG (Figure 2F). These data show that CBD and CBG differentially regulate signaling in singly transfected cells but exert a similar effect in CB_2_–5HT_1A_-Het-expressing cells.

### 2.4. CB_2_–5HT_1A_-Het Expression Was Upregulated in Glucose-Oxygen-Deprived (GOD) Primary Striatal Neurons

**Figure 3:**
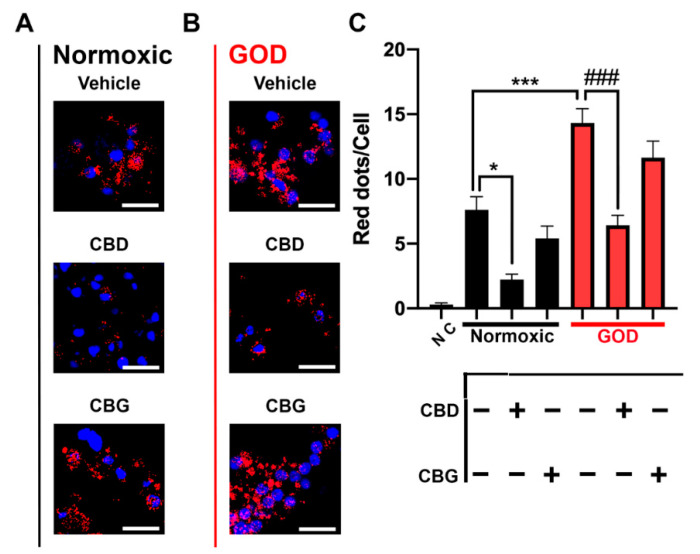
Effect of CBD and CBG on CB_2_–5HT_1A_ heteromer expression in GOD neurons. (**A**,**B**) CB_2_–5HT_1A_ heteromers were detected by in situ proximity ligation assay (PLA) in primary striatal neurons. Neurons were treated with either 200 nM CBD, 200 nM CBG, or vehicle for 30 min. Thereafter, cells were deprived of glucose and oxygen (GOD) (**B**) or were kept in aerated complete medium (**A**) for 4 h. Experiments were performed in samples from 6 different animals. Confocal images (stacks of 3 consecutive panels) were analyzed for assessing the number of red dots/cell. Red dots indicate expression of heteromers. Hoechst-stained nuclei appear in blue. Scale bar: 10 μm. (**C**) Quantification of the number of dots-clusters/cell was performed using the Andy’s algorithm Fiji’s plug-in. One-way ANOVA followed by Dunnett’s multiple comparison post hoc tests were used for statistical analysis. NC: negative control; * *p* < 0.05, *** *p* < 0.001 versus normoxic vehicle, ### *p* < 0.001 versus GOD vehicle.

Striatal neurons seeded and cultured over 12 days were labelled using the in situ proximity ligation assay (PLA, see the Materials and Methods) with specific antibodies against CB_2_ and 5HT_1A_ receptors. In complete medium and normoxia, approximately eight red dots were counted per every Hoechst-stained cell nucleus, indicating the expression of CB_2_–5HT_1A_-Hets in those neurons (Figure 3A,B). An important decrease in the receptor complex expression was observed when the same experiment was conducted in primary cultures pre-treated with CBD (approximately two red dots/cell). The effect of CBG was less marked as the number of dots per Hoechst-stained cell nucleus was around 5. Next, we investigated the expression of CB_2_–5HT_1A_-Het in GOD cells. For this, the striatal neurons were maintained for 30 min in HBSS medium without glucose and subsequently placed in an anaerobic chamber for 4 h. GOD induced an important overexpression of CB_2_–5HT_1A_ receptor complexes (around 14 red dots/cell). Once again, pretreatment with CBD and CBG induced a significant decrease in the expression of the receptor complex, (respectively, 6 and 11 red spots/cell) (Figure 3). All together, these data indicate that CB_2_–5HT_1A_-Het expression is upregulated in GOD conditions and that phytocannabinoids, especially CBD, revert the effect.

### 2.5. The CB_2_–5HT_1A_-Het Was Overexpressed in Brain Slices from Lesioned Animals

**Figure 4:**
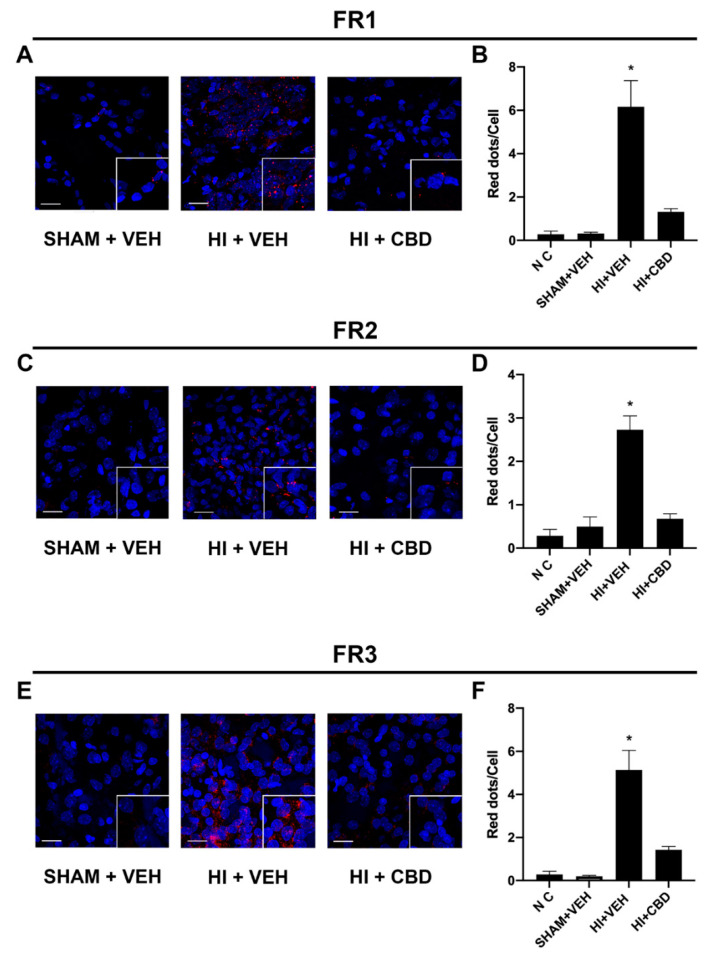
CB_2_-5HT_1A_ heteromer expression in brain slices from hypoxia-induced Wistar rats. (**A**,**C**,**E**) CB_2_–5HT_1A_-Hets expression in brain slices from hypoxia-induced Wistar rats (HI + VEH), hypoxia-induced Wistar rats treated with CBD (HI + CBD), and control Wistar rats (SHAM + VEH), detected by PLA. Rat brains were dissected 1 (FR3), 7 (FR2), or 30 (FR1) days after the insult, as described in the Materials and Methods. Red dots indicate expression of heteromers. Hoechst-stained nuclei appear in blue. (**B**,**D**,**F**) Quantification of the CB_2_–5HT_1A_Hets was conducted by detecting the number of red dots/cell using the Andy’s algorithm Fiji’s plug-in. Samples from 6 different animals were processed and analyzed. The NC negative control bar in panels B, D, and F refers to counts in cells treated only with secondary antibodies. Scale bar: 20 μm. * *p* < 0.05 versus SHAM+VEH.

Once a significant increase in the expression of the CB_2_–5HT_1A_ receptor complex was identified in a GOD cell model, PLA experiments were performed on brain slices from injured pups. Apart from the control group, two groups of lesioned animals were generated, one treated with CBD and another treated with vehicle. Pups were first exposed to carotid electrocoagulation followed with hypoxia (10% O_2_) for 112 min and treated or not with CBD. In situ PLA was first performed in cortical sections of brains taken one day after the insult. The results indicate low expression of CB_2_–5HT_1A_-Hets in control animals that underwent the same surgery without carotid electrocoagulation and that were not subjected to hypoxic conditions (SHAM) (Figure 4A). Upregulation of the receptor complex was induced by hypoxia (around six red dots/cell) and CBD was able to revert such upregulation (one red dot/cell) (Figure 4A). The expression days later after the insult was markedly decreased, showing about three and four red dots/cell in cerebral cortex sections taken, respectively, 7 and 30 days after the lesion. Once again, CBD administration led to stronger downregulation in heteroreceptor complex expression (Figure 4D,F). Cortices treated with secondary antibodies in the absence of primary antibodies showed no PLA red spots/clusters, demonstrating the specificity of the technique (Figure 4B,D,F, NC bar). Taken together, these results demonstrate an upregulation of the CB_2_–5HT_1A_-Het induced by the hypoxic insult and a significant reversal upon CBD administration.

### 2.6. CBD Abolished CB_2_–5HT_1A_-Het Functionality in GOD Striatal and Cortical Neurons

**Figure 5:**
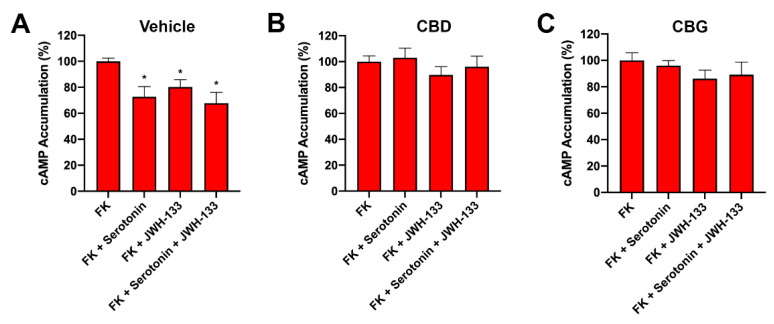
Effect CBD or CBG on the receptor functionality in GOD primary striatal neurons. Mouse primary striatal neurons were treated with vehicle (**A**), 200 nM of CBD (**B**), or 200 nM of CBG (**C**) for 30 min prior GOD for 4 h. Then, neurons were treated with selective agonists, 200 nM JWH-133, or 200 nM serotonin. cAMP levels were expressed as a percentage versus 0.5 µM forskolin treatment. Values are the mean ± S.E.M. of 8 different experiments performed in triplicate. One-way ANOVA followed by Dunnett’s multiple comparison post hoc tests were used for statistical analysis. * *p* < 0.05 versus forskolin treatment.

Finally, we addressed the effect of CBD or CBG pretreatment on the pharmacology displayed by receptors in striatal and cortical GOD neurons. In striatal neurons, G_i_ coupling was observed upon receptor activation using serotonin or JWH-133. This FK-induced lowering effect of cAMP levels after receptor activation was blocked by both CBD and CBG (Figure 5).

**Figure 6:**
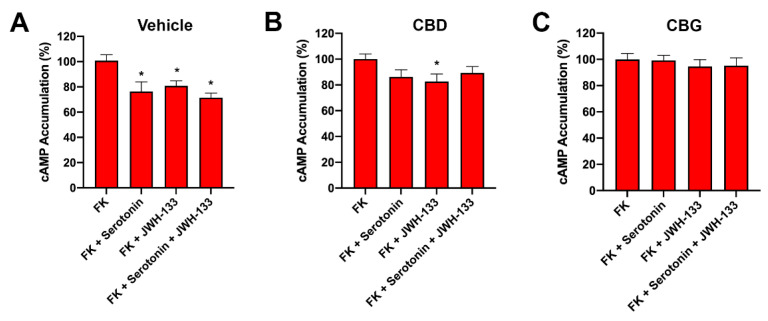
Effect CBD or CBG on the receptor functionality in GOD primary cortical neurons. Mouse primary striatal neurons were treated with vehicle (**A**), 200 nM of CBD (**B**), or 200 nM of CBG (**C**) for 30 min prior GOD for 4 h. Then, neurons were treated with selective agonists, 200 nM JWH-133 or 200 nM serotonin. cAMP levels were expressed as a percentage versus 0.5 µM forskolin treatment. Values are the mean ± S.E.M. of 8 different experiments performed in triplicate. One-way ANOVA followed by Dunnett’s multiple comparison post hoc tests were used for statistical analysis. * *p* < 0.05 versus forskolin treatment.

In similar experiments performed on primary cortical neurons, CBG blocked the cannabinoid-receptor- and serotonin-receptor-mediated effect. In the case of CBD, the effect was less noticeable, significantly blocking the effect induced by serotonin but not that exerted by JWH-133 (Figure 6).

## 3. Discussion

CBD has long been considered a neuroprotective molecule. In a previous study, it was shown in the middle cerebral artery occlusion model that CBD reduces the size of the infarcted brain area and the effect is partially blocked by WAY100135, a selective 5HT_1A_ receptor antagonist [39]. Hypoxia in the newborn can have negative consequences on the development of the nervous system. On the one hand, the sooner oxygenation is restored, the better the clinical outcome. On the other hand, it is necessary to limit the anatomical and cellular damage in the organ most susceptible to lack of oxygen, the brain. On the basis of experiments with a surrogate model of the disease, namely, the newborn piglet subjected to hypoxia-ischemia, CBD was proposed, several years ago, as an attractive drug to limit brain damage [22]. CBD is a phytocannabinoid that may interact with cannabinoid receptors; in both CB_1_ and CB_2_ receptors, the compound can enter into the orthosteric center to be a low-potency agonist and, also, it can interact with non-orthosteric sites to act as an allosteric modulator at nanomolar concentrations [14,15]. In addition, it is known that, at micromolar concentrations, CBD activates serotonin 5HT_1A_ receptors [18]; both CB_2_ and 5HT_1A_ receptors are mediators of the neuroprotection provided by CBD in an animal model of neonatal hypoxia-ischemia [25].

It has been previously shown that CB_2_ and 5HT_1A_ receptors may interact to form macromolecular complexes. The expression of such CB_2_–5HT_1A_-Hets is increased in the pig model of newborn hypoxic-ischemic brain damage. In addition, CB_2_–5HT_1A_-Het expression is tightly regulated in postnatal brain development stages; expression is relatively high at birth and declines rapidly with development of the nervous system [35]. In the rodent model used here, the increased expression of the heteromer, previously shown in the injured pig model, was reproduced and, consequently, one of the most relevant findings of this work is the significant reduction in the expression of CB_2_–5HT_1A_-Hets in CBD-treated lesioned rats. Interestingly, the previously reported upregulation of CB_2_–5HT_1A_-Hets in GOD primary neurons [35] was reversed by treating these cortical primary neurons with CBD (Figure 4).

In this study, the phytocannabinoid CBG was used in parallel with CBD because it has been suggested that the different binding modes of the cannabinoid to the CB_2_R result in different output signals. A previous report addressed how the different CBD and CBG-type phytocannabinoids behave with respect to the functionality of cannabinoid CB_1_ and CB_2_ receptor. The results showed that it is the binding mode that makes the functional response vary from phytocannabinoid to phytocannabinoid [11]. It is tempting to speculate that differential benefits of phytocannabinoids in terms of therapeutic potential could depend on the binding mode, i.e., on how each molecule interacts with the orthosteric site and with exosites in the CB_2_R. This is particularly relevant when it comes to cannabinoid receptors since (i) orthosteric sites have room accommodate different structures differently, (ii) orthosteric sites are not open to the extracellular medium, (iii) entry to the orthosteric center occurs through the lipid bilayer of the membrane, and (iv) the entrance is constituted by a narrow vestibule in which part of the chemical structure can be trapped [11,40,41,42,43]. Our results on comparing CBD and CBG effects are consistent with differences in binding modes that may be further modulated due to allosteric modulations resulting from the interaction of the 5HT_1A_ receptor with the CB_2_ receptor. However, the differences found in cells expressing only one of the receptors were markedly reduced in cells expressing CB_2_–5HT_1A_-Hets. It is also true that the effect of CBG on the regulation of CB_2_–5HT_1A_-Het expression in primary cortical neurons was much weaker than that exerted by CBD.

The similar effect of CBD and CBG on primary GOD neurons opens the way to the hypothesis that in a hypoxia-ischemia environment, serotonin is harmful. Given that both phytocannabinoids blocked the effect of serotonin and there is consensus on the benefits of CBD in models of neonatal hypoxia, suppression of 5HT_1A_ receptor-mediated signaling may be beneficial. This hypothesis would fit with the need to reduce the expression of CB_2_–5HT_1A_-Hets shortly after birth for proper brain development. It would be good to assess the potential of 5HT_1A_ receptor antagonists in GOD neurons or hypoxia-ischemia models. At present, this possibility is hampered by the fact that most of the antagonists of 5HT_1A_ receptor, e.g., alprenolol, may also interact with adrenergic receptors [44,45]. To our knowledge, there are no studies on the direct effect of more selective 5HT_1A_ receptor antagonists, e.g., spiroxatrine or WAY100135, in models of stroke or hypoxia-ischemia. There is, however, a report showing benefits of antagonizing the 5HT_1A_ using WAY100135 in a rodent model of intestinal ischemia-reperfusion [46].

## 4. Materials and Methods

### 4.1. Reagents

JWH-133 and serotonin were purchased from Tocris Bioscience (Bristol, UK). Coelenterazine H was purchased from Molecular Probes (Oregon, USA), and forskolin was purchased from Sigma-Aldrich (Missouri, US). Purified (>95% pure) cannabinoids were provided by Phytoplant Research S.L.U, Córdoba, Spain. Preparations of CBD were purified from the Cannabis variety GOYA (CPVO 20180113) and CBG, obtained from the variety AIDA (CPVO/20160167) following a direct crystallization method (Nadal, 2016; patent US9765000 B2 and WO2016116628A1), which allows compounds with a purity > 98% to be obtained.

### 4.2. HI Brain Damage Induction

Experimental procedures in rats were conducted in accordance with European and Spanish regulations (2010/63/EU and RD 53/2013) and approved by the Institutional Review Board of Hospital Clínico San Carlos-IdISSC (Madrid, Spain, protocol code ProEx 165/19, date of approval 25 February 2019). HI brain damage protocol is elsewhere described (Pazos et al., 2012). In brief, 7- to 10-day-old (P7–P10) Wistar rats were anesthetized with sevoflurane (5% induction, 1% maintenance). Exposed left carotid artery was electrocoagulated and, after recovery (a 3 h), pups were placed for 112 min into 500 mL jars in a water bath (37 °C) in 10% O_2_. Control animals undertook the same surgical procedure but skipping electrocoagulation and hypoxia (SHAM). Ten minutes after the end of hypoxia, HI pups were randomly treated with s.c. injection of vehicle (HI + VEH, *n* = 27) or CBD (HI + CBD, *n* = 29). CBD was injected at a dosage of 1 mg/kg in 0.1 mL final volume. Then, rats were returned to the dam. On the day of the sacrifice, a T2WI MRI scan of the brains was carried out in the MRI Unit of the *Instituto Pluridisciplinar* (*Universidad Complutense de Madrid*, Madrid, Spain) on a BIOSPEC BMT 47/40 (Bruker-Medical, Ettlingen, Germany) operating at 4.7 T to determine the volume of damage, as described in detail elsewhere [24,25,26]. The rats were sacrificed 1 (FR3), 7 (FR2), or 30 (FR1) days after challenge, and the brains were removed and processed as described below.

### 4.3. Brain Sampling

Rats under deep anesthesia (i.p. injection of diazepam/ketamine) were sacrificed. Perfusion was performed transcardially with saline solution and 4% paraformaldehyde. Brains were removed to be embedded in paraffin. Coronal sections (30 µm thick) using a cryostat LEICA CM3050 S (Leica Microsystems, Wetzlar, Germany) were obtained for the immunohistochemical/PLA assays.

### 4.4. Cell Culture and Transfection

Human embryonic kidney HEK-293T (lot 612968) cells were acquired from the American Type Culture Collection (ATCC, Manassas, VA, USA). Each frozen aliquot was thawed, and the cells it contained were passaged 18 times before a new aliquot was taken. Culture medium was Dulbecco’s modified Eagle’s medium (DMEM) (Gibco, Waltham, MA, USA) supplemented with 2 mM L-glutamine, 100 U/mL penicillin/streptomycin, MEM non-essential amino acid solution (1/100), and 5% (*v/v*) heat-inactivated fetal bovine serum (FBS) (all supplements were from Invitrogen, Paisley, Scotland, UK). Cultures were kept in 5% CO_2_ humid atmosphere (37 °C). Cells were transiently transfected using the PEI (PolyEthylenImine, Sigma-Aldrich, St. Louis, MO, USA) method as previously described [47,48]. At 4 h after transfection, growth medium was replaced by complete medium. Experiments were carried out 48 h later.

### 4.5. Neuronal Primary Cultures

Neurons from the brain of fetuses (gestational age: 17 days) of pregnant CD1 mice (14–18 weeks old) were isolated as described elsewhere [49]. No ethical approval is needed for this protocol as long as no distinction is made between male/female sex of fetuses. Cells were plated at a confluence of 40,000 cells/0.32 cm^2^. After trypsinization, cell suspension was repeated pipetted up and down followed by passage through a 100 μm pore mesh. Centrifugation (7 min, 200 × *g*) led to a pellet of cells that were resuspended in complete DMEM and seeded in 6-well plates at a density of 3.5 × 10^5^ cells/mL. Then, 24 h later, the medium was replaced by neurobasal medium supplemented with 2 mM L-glutamine, 100 U/mL penicillin/streptomycin, and 2% (*v/v*) B27 medium (Gibco). Neurons were cultured for 12 days before assays. The use of NeuN allowed us to know that >90% cells in the culture were neurons.

### 4.6. Expression Vectors

The human cDNAs for the CB_2_ and 5HT_1A_ receptors cloned in pcDNA3.1 were amplified using sense and antisense primers that were designed to eliminate stop codons. The primers harbored either unique EcoRI and BamHI sites to clone CB_2_ and GHS-R1a receptors were subcloned to a pEYFP-containing vector to be in frame with a yellow fluorescent protein (pEYFP-N1; Clontech, Heidelberg, Germany). Primers harboring unique KpnI and BamHI and sites for β-arrestin 2 and 5HT_1A_R were subcloned to the pRLuc-N1 vector (PerkinElmer, Wellesley, MA, USA) to obtain a plasmid containing the sequence of a fusion protein with the Renilla luciferase protein (RLuc). A similar procedure was used to have fusion proteins with pEYFP. The generated constructs were CB_2_R-YFP, 5HT_1A_R-YFP, 5HT_1A_R-RLuc, GHS-R1a-YFP, and β-arrestin 2-RLuc.

### 4.7. Glucose-Oxygen Deprivation (GOD)

Twenty-four hours prior to assay performance, cell medium was exchanged by glucose-free HBSS medium and treated with 200 nM CBD, 200 nM CBG, or vehicle to subsequently establish normoxic conditions (37 °C and 5% CO_2_ atmosphere). Conditions were maintained for 30 min prior to placing cells in an anaerobic chamber (AnaeroPack Rectangular Jar 2.5 L; Thermo Scientific, Waltham, MA, USA) for 4 h with an anaerobic atmosphere-generator bag (AnaeroGen 2.5 L; Thermo Scientific, Waltham, MA, USA).

### 4.8. Immunofluorescence

HEK-293T cells transfected with cDNAs for CB_2_R-YFP and 5HT_1A_R-RLuc were fixed in 4% paraformaldehyde for 15 min and then washed twice with PBS containing 20 mM glycine. A 0.2% Triton X-100 solution in the same buffer was used for permeabilization (5 min incubation). After 1 h in blocking solution (PBS containing 1% bovine serum albumin), cells were incubated with a mouse anti-RLuc antibody (1/100; MAB4400, Millipore, Burlington, MA, USA) and a secondary Cy3-conjugated anti-mouse IgG (1/200; 715-166-150; Jackson Immuno Research). After washing, samples were treated with mounting media (30% Mowiol; Calbiochem, San Diego, CA, USA). Nuclei were stained with Hoechst (1/100). A Zeiss 880 confocal microscope (Leica Microsystems, Wetzlar, Germany) was used for obtaining images.

### 4.9. Bioluminescence Resonance Energy Transfer (BRET) Assay

HEK-293T cells growing in 6-well plates were transiently co-transfected with two of the plasmids described in Section 2.6. Then, 48 h post-transfection, cells were washed twice with 0.1% glucose (*w/v*) in HBSS (137 mM NaCl, 5 mM KCl, 0.34 mM Na_2_HPO_4_, 0.44 mM KH_2_PO_4_, 1.26 mM CaCl_2_, 0.4 mM MgSO_4_, 0.5 mM MgCl_2_, and 10 mM HEPES; pH 7.4). After detachment by gentle pipetting, the cells were resuspended in the same buffer. Protein concentration was determined using a Bradford assay kit (Bio-Rad, Munich, Germany) and bovine serum albumin dilutions as standards. YFP-fluorescence was determined in 96-well black plates with a transparent bottom (Porvair, Leatherhead, UK) using a FluoStar Optima fluorimeter (BMG Labtechnologies, Offenburg, Germany) equipped with a high-energy xenon flash lamp, reading at 530 nm. Data are given as the fluorescence (20 μg protein in each sample) minus the fluorescence of cells only expressing protein-RLuc. BRET measurements were made using white 96-well plates (Porvair); in each well, a suspension (20 μg protein) of cells treated or not with cannabinoids was placed. Pretreatments were 30 min using 200 nM CBD, 200 nM CBG, or vehicle. Recordings began after the addition of 5 μM coelenterazine H (Molecular Probes, Eugene, OR). The BRET reader was a Mithras LB 940 (Berthold, Bad Wildbad, Germany), allowing integration of signals detected on the long wavelength filter at 530 nm (520–540 nm) and on the short wavelength filter at 485 nm (475–495 nm). To quantify receptor-RLuc expression, luminescence readings were collected 10 min after 5 μM coelenterazine H addition. The net BRET is defined as [(long-wavelength emission)/(short-wavelength emission)]-Cf, where Cf corresponds to [(long-wavelength emission)/(short-wavelength emission)] for the RLuc construct expressed alone in the same experiment. The BRET curves were fitted by non-linear regression. BRET values are given as milli BRET units (mBU: 1000× net BRET).

### 4.10. β-Arrestin 2 Recruitment

β-Arrestin 2 recruitment was determined as previously described [14] in cells transfected with one or more of the plasmids described in Section 2.6. Cells (20 μg protein) were distributed in 96-well white plates with a white bottom (Corning 3600) and incubated with compounds (see figure legends) for 10 min before the addition of 5 μM coelenterazine H. Then, 1 min after coelenterazine H addition, BRET was determined in a Mithras LB 940. To quantify protein-RLuc expression, luminescence was measured 10 min after the addition of 5 μM coelenterazine H.

### 4.11. cAMP Determination

The ad hoc LanceUltra kit (PerkinElmer, Waltham, MA, USA) was used for cAMP determination using homogenous assays. Transfected HEK-293T cells or primary neurons were seeded in 6-well plates. Two hours before initiating the experiment, culture medium was substituted by non-supplemented DMEM medium. After detachment, cells were re-suspended in non-supplemented medium containing 50 μM zardaverine. Cells were pretreated (30 min) with 200 nM CBD, 200 nM CBG, or vehicle and, 5 min later, stimulated with selective agonists. Forskolin (0.5 μM) or vehicle were then added for a period of 15 min. Finally, the reaction was stopped by the addition of the Eu-cAMP tracer and the ULight-cAMP monoclonal antibody prepared in the “cAMP detection buffer” of the LanceUltra kit. All steps were performed in 384-well microplates at 25 °C. Then, 60 min later, homogeneous time-resolved fluorescence energy transfer (HTRF) measures were obtained in a PHERAstar Flagship microplate reader equipped with an HTRF optical module (BMGLab technologies, Offenburg, Germany).

### 4.12. Proximity Ligation Assay (PLA)

Physical interaction was detected using the Duolink in situ PLA detection kit (Duolink, St. Louis, MO, USA) following the instructions of the supplier. Cells placed on glass coverslips or fixed brain sections were washed with PBS containing 20 mM glycine to quench the aldehyde groups; 0.05% Triton X-100 in the same buffer (20 min) was used for permeabilization. After 1 h at 37 °C with blocking solution, primary cultures were incubated overnight with a mixture of equal amounts of mouse anti-CB_2_R (1/100; sc-293188, Santa Cruz Technologies, Dallas, TX, USA) and rabbit anti-5HT_1A_R (1/100, ab85615, Abcam, Cambridge, UK) antibodies to detect CB_2_R–5HT_1A_R complexes. Neurons were processed using the PLA probes that detect primary antibodies (Duolink II PLA probe plus and Duolink II PLA probe minus) diluted in the antibody diluent solution (1:5). Ligation and amplification were performed as indicated by the supplier. Hoechst (1/100; Sigma-Aldrich) was used to stain nuclei. For negative control, cells were treated with secondary antibodies in the absence of primary antibodies. A Zeiss 880 confocal microscope (Leica Microsystems, Wetzlar, Germany) equipped with an apochromatic 63× oil immersion objective (N.A. 1.4) and 405 and 561 nm laser lines was used for getting images. In each observation, data corresponding to a stack of two channels (one per staining) and to four Z stacks with a step size of 1 μm were acquired. Data analysis was performed using the Andy’s algorithm Fiji’s plug-in. One-way ANOVA followed by Dunnett’s multiple comparison post hoc tests were used for statistical analysis.

### 4.13. Data Handling and Statistical Analysis

Data were analyzed blindly. Data are presented as the mean ± SEM. Statistical analysis was performed with SPSS 18.0 software. The test of Kolmogorov–Smirnov with the correction of Lilliefors was used to evaluate normal distribution and the test of Levene to evaluate the homogeneity of variance. Significance was analyzed by one-way ANOVA, followed by Bonferroni’s multiple comparison post hoc test. Significance was considered when *p* < 0.05.

## Data Availability

Data that may be eventually missing can be obtained from the corresponding author upon reasonable request.
